# Emerging Environmental Justice Issues in Nuclear Power and Radioactive Contamination

**DOI:** 10.3390/ijerph13070700

**Published:** 2016-07-12

**Authors:** Dean Kyne, Bob Bolin

**Affiliations:** 1 Department of Sociology and Anthropology, the University of Texas Rio Grande Valley, Edinburg, TX 78539, USA; 2 School of Human Evolution and Social Change, Arizona State University, Tempe, AZ 85287, USA; bob.bolin@asu.edu

**Keywords:** environmental justice, U.S. commercial nuclear power plants, radioactive contamination, nuclear weapons, uranium mining, American Indians, nuclear energy ethics

## Abstract

Nuclear hazards, linked to both U.S. weapons programs and civilian nuclear power, pose substantial environment justice issues. Nuclear power plant (NPP) reactors produce low-level ionizing radiation, high level nuclear waste, and are subject to catastrophic contamination events. Justice concerns include plant locations and the large potentially exposed populations, as well as issues in siting, nuclear safety, and barriers to public participation. Other justice issues relate to extensive contamination in the U.S. nuclear weapons complex, and the mining and processing industries that have supported it. To approach the topic, first we discuss distributional justice issues of NPP sites in the U.S. and related procedural injustices in siting, operation, and emergency preparedness. Then we discuss justice concerns involving the U.S. nuclear weapons complex and the ways that uranium mining, processing, and weapons development have affected those living downwind, including a substantial American Indian population. Next we examine the problem of high-level nuclear waste and the risk implications of the lack of secure long-term storage. The handling and deposition of toxic nuclear wastes pose new transgenerational justice issues of unprecedented duration, in comparison to any other industry. Finally, we discuss the persistent risks of nuclear technologies and renewable energy alternatives.

## 1. Introduction

Nuclear technologies, both from military and commercial applications, pose a complex of environmental justice issues in terms of current and future risks they pose to people and environments. These risks, as both nuclear testing and nuclear reactor accidents have shown, transcend national boundaries, can span millennia, and can have multigenerational health risks. To explore nuclear risks and environmental justice, we focus on issues only in the continental U.S. However, we recognize that the risks are expanding at a global level with accumulations of high-level nuclear waste, mining and milling waste, and unresolved serious nuclear contamination problems at military sites in North America, Europe, Africa, and Asia (see [1]).

Weapons programs and nuclear power generation involve a complex nuclear fuel cycle, from mining and milling of the ore, to enrichment and nuclear fuel rod or warhead fabrication, leaving at the end of the cycle extremely toxic high-level nuclear waste that must be isolated from humans for hundreds of thousands of years [2]. This complex and geographically dispersed fuel cycle instantiates multiple exposure pathways, persistent risks, and growing questions about safety over time-spans beyond any human experience. While the more than 900 nuclear detonations in the continental U.S. alone have amply demonstrated the massive destructive power and toxic fallout potential of nuclear weapons, commercial nuclear power has also proved repeatedly to lack the “absolute safety” guaranteed by industry proponents (see [3]). As a series of catastrophic reactor accidents has shown, the commercial uses of nuclear fission materials to generate electricity are not without potentially severe multiscale risks. (Our discussion focuses solely on commercial reactors. There are dozens of research reactors in the U.S., both in federal nuclear research facilities and at other locations. Research reactors have had notable and very serious contamination events although they are outside our purview in this paper (see [4]). This potential has been variously displayed at Chernobyl in Russia (1986), Three Mile Island (TMI) in U.S. (1979), and most recently at Fukushima in Japan (2011). Indeed, on the 30th anniversary of the Chernobyl disaster (2016), no technology yet exists to handle the melted highly radioactive 2000-ton core of the failed reactor, a core that will be lethal to humans for thousands of years [5]. 

Beyond the catastrophic failures, even in routine operations, nuclear power plants (NPPs) are a source of low level ionizing radiation potentially affecting workers and those living in proximity to reactor sites, particularly children (for a review or research in the European context (see [6]). A growing concern of those critical of the U.S. nuclear industry is that the aging and deteriorating U.S. fleet of reactors may be at increased risk of leaks, unplanned releases, and other failures given that most are already operating beyond their original 40-year operational life [7]. When considering the environmental justice implications of nuclear technologies it is critical to focus on the entire cycle from ‘cradle to grave’ of nuclear materials and technologies as this calls attention to the full spatial and temporal expressions of the nuclear hazardscape, something proponents fail to do when touting the putative zero carbon emissions of NPPs.

The toxic legacies of the global nuclear weapons complex—from uranium mining and milling, to weapons production and testing—have been global in reach, from Kyrgyzstan to the Marshall Islands to the Navajo Nation and Alaska [1,8]. Radioactive contamination of civilian populations in the U.S. (and globally) began with above-ground nuclear weapons tests at the Nevada Test Site (NTS) in 1951 producing the first “downwinders”—unprotected civilian populations exposed to nuclear fallout [9]. (Prior to surface testing in the U.S., there was an extensive testing program in the U.S. Marshall Islands, and thousands of Pacific Islanders were displaced and suffered radiation exposure (see [1]). Entire islands were vaporized by hydrogen bombs and islanders, decades later, remain displaced from home islands too contaminated to occupy. Today, those living near contaminated sites making up the U.S. weapons complex continue to be exposed to environmental health risks relating to radiation and related chemical contaminants [10]. The fact that many of those exposed to U.S. (and international) nuclear weapons production and testing are colonized indigenous groups and racialized minorities, raises clear environmental justice concerns [1]. The geographical scale and costs of the U.S. nuclear industrial complex exceeds virtually that of any other industrial sector (estimates place the total costs over 60 years of producing weapons grade materials, manufacturing, testing, maintaining, and decommissioning some 70,000 nuclear weapons at approximately $7.5 trillion as of 2005 (p. 47 [11]). The weapons complex also occupies (and contaminates) 36,000 square miles of the U.S., much of it federal sites on public lands in proximity to Indian reservations and other population centers [12]. 

To approach the topic, our discussion is organized into four sections. First we examine distributive and procedural justice issues at commercial reactors in the U.S. Our concern here is to examine the nature of the at-risk populations living in proximity to civilian nuclear power plants in the U.S. and discuss some of the environmental risks and procedural justice issues that pertain to nuclear plant siting, license renewal decision-making, and emergency preparedness. We highlight the growing constraints by the Nuclear Regulatory Commission (NRC) in public participation in nuclear decision-making, which appear to reinforce a tradition of secrecy, denial, and misinformation that has long been part of the nuclear industrial complex [1,13]. The second part of this paper addresses Cold War radiation exposure legacies and their environmental justice implications. We discuss the diverse pathways of radiation exposure for residents of Western states as a result of nuclear weapons testing fallout and from exposure to the extensive mining, milling, and weapons manufacturing processes that have contaminated communities across the American West [14]. In the next section, to illustrate the persistent risks of nuclear weapons production, we briefly discuss two federal nuclear weapons centers—Los Alamos, NM and Hanford, WA—as exemplars of the complex problems in handling nuclear waste and in reducing risks to surrounding communities.

The fourth section of the paper discusses the issue of safe disposal of high-level nuclear waste from power plants and weapons production. As of this writing no geologic burial site is open and operational that can provide secure storage of nuclear waste in the U.S. Further, there is a great deal of citizen opposition to the transportation of highly radioactive waste through cities and towns to secure storage sites should they become available in the future [15,16,17]. We conclude the paper with a discussion of persistent safety and environmental health issues in the nuclear complex and briefly discuss alternative energy sources for reducing nuclear risks and enhancing long-term environmental sustainability and justice.

## 2. Environmental Justice Issues in Commercial Nuclear Power

When discussing environmental justice issues we are concerned with three key forms of justice. The first, distributive justice, refers to how environmental burdens are sociospatially distributed, and what principles are used in allocating risks [18]. The second is procedural justice; that is, how equitable or inequitable the processes are whereby decisions are made regarding the impositions of environmental risks on people and places [2]. Lastly, recognition justice concerns who is or is not recognized as worthy of inclusion in decision-making regarding the allocation of hazard burdens. This is particularly salient in looking at nuclear injustice in American Indian communities, where native lands were declared “wastelands” by the military, thus failing to recognize people who have occupied landscapes for millennia [19]. 

We begin by considering distributive justice issues in U.S. NPPs. In general, individuals living around nuclear power plants face potential health risks posed by complex nuclear technologies. There are two categories of risks: those stemming from day-to-day operations and those arising from catastrophic failures. In case of acute failures, large areas around the nuclear plants face potential exposure to highly toxic radioactive releases, soil and water contamination, radiation from melted fuels, and large exclusion zones of uninhabitable land (as in both Chernobyl and Fukushima). The Fukushima Daiichi nuclear disaster in 2011 is the most recent reminder that nuclear power plants are subject to catastrophic failures with the potential to produce radiation-related diseases, as well displace hundreds of thousands of people and render large areas contaminated for centuries. And while these extreme events are relatively infrequent, when they occur multiple generations will be burdened with the environmental and health costs of these disasters, as Chernobyl has amply demonstrated (see [20]). 

Reactors pose environmental and health risks even during routine operation in the form of low level radioactive emissions from a variety of sources [21]. Further, with the U.S. commercial nuclear reactor aging, concerns exist that the likelihood of cooling system leaks, contamination events, plant fires, and other “normal accidents” could increase in frequency with aging and degrading plant infrastructure [7,22]. Individuals living near nuclear power plants are potentially exposed to various sources of ionizing radiation. Every reactor releases radioactive gases that are routinely vented through stacks in the reactor roof and from the steam generators; every hour about 100 cubic feet of radioactive gases are released; purging of radioactive materials in pipes is conducted frequently (22 purges per year are allowed per reactor); discharging radioactive water into surrounding areas when it is too hazardous for plant workers to handle; using 20,000 gallons of water for cooling the reactor core every minute, with the cooling water becoming contaminated by radioactive tritium (tritiated water). Of this, 5000 gallons of tritiated water per minute are released into adjacent lakes, rivers, or the ocean, and an additional 15,000 gallons are vented into the atmosphere as steam [20]. (The potential health effects of exposure to radionuclides include (1) tritium or tritiated water becoming a part of bodily fluids within one or two hours of exposure; (2) plutonium-23 causing blood cancers such as lymphoma or leukemia; (3) iodine-131 which is quickly absorbed by the thyroid causing thyroid cancer; (4) strontium-90 which the body treats like calcium staying in the breast causing breast cancer; (5) Cesium-137 which is absorbed by muscle cells causing cancer; and (6) radioactive noble gases causing mutations in eggs and sperm [23]).

The World Nuclear Association claimed that it is difficult to detect the cancer in the individuals who are exposed to less than 100 mSv [24]. The U.S. NRC has also claimed that biological effects from exposure to low level radiation are small and may not be detectable [25]. The U.S. Environmental Protection Agency (EPA) provided guidelines to evacuate or remain in shelter when the radiation dose reaches between 1 and 5 rems (10 mSv to 50 mSv) projected dose over four days in the early stage of nuclear power accident [26]. Nevertheless, in the past 30 years, scientists in Europe and the USA have repeatedly studied and confirmed that normal operation of reactors causes cancer, especially in children [27,28,29,30,31,32,33,34,35,36,37,38,39,40,41,42,43,44,45,46]. The radiation releases during the Fukushima, Chernobyl, and TMI accidents were much higher than permitted releases during normal operation [27]. Further, the U.S. National Academy of Sciences claims that there is no safe dose of ionizing radiation and even normal background radiation can cause cancer [47,48]. 

Given a host of routine radiation risks that plants pose, in this section we discuss the ways that communities near nuclear power plants face environmental injustice issues from the disproportionate risk burdens they bear. To better understand these environmental justice issues, we examine NPP host communities from both distributive and procedural justice perspectives, examining locational and proximity issues as they relate to plant siting decisions, emergency preparedness, and public participation in nuclear energy decision-making.

### 2.1. Distributive Justice—Locational and Proximity Issues

**Figure 1:**
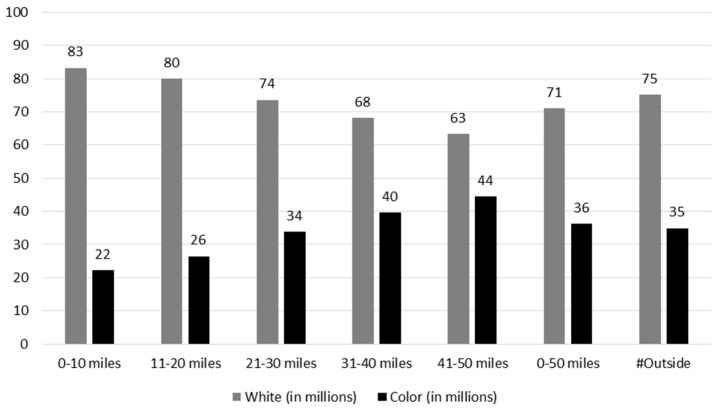
Demographic Composition of Percent white and color according to area, sorted by distance from any one of the 61 commercial NPPs, based on 2010 American Community Survey data.

In this section we compare populations living within a 50-mile radius of plant sites to the population residing beyond that perimeter for all operating plants in the U.S. The 50-mile radius conforms to the NRC’s Emergency Planning Zone (EPZ) Ingestion Pathway, the outer geographic limit of NRC planning for nuclear contamination events. Kyne [49] conducted a study of distributive justice based on 104 reactors at 65 sites. In this study, using the same dataset (namely the U.S. Census 2010/American Community Survey, 5-year estimate) [50] and adopting the same methodology (see [49]), we estimated distribute justice around the current 99 operational reactors at 61 sites in 31 states. Six reactors have been shuttered for various reasons since Kyne’s previous study. The dataset that results from each respective survey include racial and ethnic subgroups, white-alone, Hispanic-alone, American Indian- or Alaskan Native-alone, Asian-alone, black or African American-alone, Native Hawaiian or Other Pacific Islander-alone and Other-alone—as well as one additional category, “Two or more races”, that is included only in the 2010 dataset. Based on the most recent census, there were approximately 87.5 million people living within a 50-mile radius of plants (Table 1, Figure 1). Looking at each racial and ethnic group, the total population was 71% white, while 36.32% were in the “Color” category. Approximately four percent more whites reside outside EPZs than inside communities than reside inside (75% vs. 71%). In contrast, a larger percentage of African Americans live within the 50-mile zone than reside outside it (17% vs. 10%) as shown in Table 1. In contrast to African Americans, fewer Hispanics are found in the host communities than outside (13.2% vs. 16%). Similar findings are observed for other racial groups as shown in Table 1. While these differences are relatively small compared to asymmetries noted in other hazardous sites, the larger issue is potential exposure to a very large numbers of residents in the case of a catastrophic failure.

To examine potential exposure of an urban population to a highly toxic radioactive plume from a reactor core breach, we use the Palo Verde Nuclear Generating Station (PVNGS) near Phoenix as an example. The reactor complex lies approximately 50 miles west and upwind of central Phoenix. Using NRC software to model the dispersion of a radioactive plume, using typical early spring wind conditions, we found 700,000 residents would be exposed out of the 4 million population in the metropolitan Phoenix area (typifying weather conditions in January and March include prevailing wind direction of west-northwest, at a speed of 6.2 miles/h) [51]. The estimated exposed population could have been even larger, but the NRC software used to model the dispersion plume is limited to a 100-mile dispersal zone (the simulation utilized the Nuclear Regulatory Commission’s Radiological Assessment System for Consequence Analysis (RASCAL) Source Term to Dose (STDose) software to project the plume pathway and radiation dose). By comparison, the Indian Point New York generating station lies within 25 miles of New York City, the most populous city in the U.S. Here a failure could generate cleanup costs of $1 trillion in the event of a core meltdown [52].

Evidence suggests that individuals living near the nuclear power plants face difficult-to-avoid health risks associated with exposure to low level routine radioactive effluents emitted from plants. Given that no level of radiation exposure is considered safe, any excess exposure could have deleterious impacts on human health [6]. The effects of radiation at the cellular level could lead to irreversible damage and potential premature death. Tritium, to highlight a common isotope, is a carcinogen, mutagen, and teratogen and can easily be incorporated into human tissues causing cancers, chromosomal aberrations, birth defects and miscarriages, and mental retardation after in utero exposure [6]. We observed that among the estimated 87.5 million people living within a 50-mile radius of a NPP (Table 1), 5.6 million (6.4%) are children under the age of five years. Children have been found to be particularly vulnerable to radiation exposure as European studies on leukemia have found. A study in Germany reported that the children under five years of age living within a 5 km (3.1 miles) are 2.19 times more likely to develop leukemia [53] than those outside this zone. And while such findings are still debated (e.g., [54]) many are strongly convinced by the evidence (e.g., [55]).

### 2.2. Procedural Justice Issues—Plant Siting, Emergency Preparedness, Public Participation, and Nuclear Energy Ethics

In addition to the 50 mile EPZ, the NRC also designates a plume exposure pathway zone within a 10-mile radius of a reactor [56]. While the large numbers of people residing within the 50-mile EPZ raises obvious hazard exposure and distributional justice issues, and procedural justice issues also confront adjacent communities. Key procedural justice issues include the nuclear power plant site selection process, emergency preparedness capabilities, and public participation in nuclear power plant license renewal procedures.

**Plant siting:** The Atomic Energy Commission (AEC) acted as a sole responsible authority for the site selection process in the beginning years of civilian nuclear power (1957–1975) [57]. Under the AEC’s guidelines there were three key siting criteria—an exclusion area, a low-population zone, and distance to major population centers [49,57]. The exclusion area was a circular zone of a size defined by the licensee, which was in turn surrounded by a low-population zone. Residential land use was not permitted in the exclusion area, and the adjacent low-population zone was to have a population of a size that could easily be evacuated in the event of a serious accident [57]. The AEC guidelines were ambiguous at best, lacking any specification of the exclusion area radius, no quantified limit on the population size for the low-population zone, and a lack of a quantified population size to be used to define the nearest densely populated area. The three key terms lacked clarity and specificity in the AEC’s siting decision-making [49,57]. Given that the EJ movement was years in the future, the AEC’s site selection and licensing decision-making also lacked any consideration of social equity in plant location. 

The Nuclear Regulatory Commission was created by the Energy Reorganization Act of 1974, replacing the much criticized AEC [58]. The act authorized the NRC to be the sole authority for the licensing of all US nuclear power reactors, for both construction and operating license applications, in a two-step process [49,59]. According to existing law, mandatory public hearings are scheduled in the licensing process, in which the public could raise their concerns and issues related to the plant’s design and construction activities that could negatively impact their health [49,60]. However, by the time the NRC was created, 81 out of the current 99 nuclear reactors had already been licensed for construction, meaning the effects of public participation would at best be limited to new plants [61]. The agency reorganized the two step licensing process into a single step in 1992, which combined the construction and licensing processes [60]. The single step process has been criticized for discouraging public participation by reducing the number of public hearing meetings and imposing the requirement for legitimate contentions for public hearings normally adjudicated by the Atomic Safety and Licensing Board (ASLB). This consists of a three-judge panel of NRC employees, made up of two technical experts and one attorney [49,60]. A limited window of 60 days to contest licensing and construction and high costs (an estimated costs of between $100,000 and $500,000 for a given case) associated with attorney and nuclear expert fees limit the public’s ability to intervene in the process [49,60]. At a time when environmental justice activism has been demanding a greater role for public participation in environmental decision-making, the NRC has acted to further constrain public involvement. 

**Emergency** **preparedness:** Critical in insuring public safety in the event of a nuclear disaster is robust emergency planning and a coherent response strategy by the reactor operator and the state. As the Fukushima Daiichi disaster has recently showed, the lack of emergency preparedness by the commercial plant operator, inadequate communication pathways, and various dysfunctions among government agencies can move a nuclear disaster rapidly toward a worst case scenario [3]. When there is an emergency at a nuclear power plant, any call for evacuation requires a long complex procedure, one that is unrealistic in the face of what actually transpires in a nuclear emergency. For an evacuation order to be issued, a computer model has to be run projecting a fallout path, protective actions have to be recommended by state authorities, and then an evacuation order issued. According to NRC [62], an extraordinarily unrealistic 15-min time frame is provided for this complex assessment process and transmitting an evacuation recommendation from the power plant operator to the state authorities [51]. There is good evidence that nuclear plant safety and security needs substantial improvement in the U.S. and that preparedness is weak for handling a nuclear emergency, particularly a rapidly evolving one that involves a cascade of reactor failures such as Fukushima (see [3]). 

**Public** **participation:** While public participation and the right to know has been a hallmark of the environmental justice (EJ) and anti-toxics movements, much of the nuclear industry has been shrouded in secrecy and public exclusion [13,63]. Based on the Atomic Energy Act, the NRC has been authorized to issue licenses to NPPs to operate up to 40 years and allows plants to be renewed for another 20 years [64]. On paper, the general public is encouraged to participate in the NRC decision-making process through public meetings, and public comment periods on rules, renewal guidance, and other documents [65]. Nevertheless, 97 out of 99 U.S. commercial nuclear power reactors have had their licenses renewed for another 20 years [61], which suggests that most renewals are pro forma given the substantially different ages of plants and their operational histories [7]. 

According to Executive Order (E.O.) 12898, issued in 1994 [66], federal agencies are mandated to identify and address adverse human health and environmental impacts on minority and low-income populations. However, it is not mandatory for independent federal agencies such as NRC. The NRC has stated that the agency has voluntarily committed to undertake environmental justice assessments during the mandated supplemental environmental impact assessments (SEIS) for license renewal [67]. In the SEIS, a number of factors are evaluated including air quality, water use, ecosystem effects, and various health and socioeconomic issues. For example, in the case of the Palo Verde Nuclear Generating Station Arizona (PVNGS) license renewal process, there were more than 90 separate issues considered. It is at the NRC’s discretion to decide how significant each of the issues are, and not surprisingly 76 percent were labeled as being of ‘small’ significance. Notably, human health and environmental justice were labeled as ‘uncertain,’ meaning no action was taken on them in the absence of adequate information.

The license renewal procedure touches on the issue of procedural justice specifically as it relates to public participation. In this case the license renewal process began on 15 December 2008 when the PVNGS submitted the application and it ended on 22 April 2011 when a decision was reached. While the process took about 44 months, the public had only two opportunities for involvement, once in 2009 and again in 2010. The NRC announced the public meetings in the Federal Register [67] 30 days before the meeting and as a result public participation was marginal. Out of a potentially affected population of 690,000 (in the 50-mile EPZ), 12 citizens provided comments. Further, the NRC, acting as a promoter of nuclear power, rebutted public comments about negative environmental impacts and dangers of aging reactors. The long troubled nuclear plant which has been under the scrutiny of the NRC due to some significant structural issues was nevertheless granted an extension of 20 years on its license rather than being closed due to safety concerns. 

**Nuclear** **energy** **ethics**: The three ethical aspects related to nuclear power are risk, justice, and democracy [68]. To elaborate nuclear energy ethics, a case study of Pilgrim Nuclear Power Station license renewal is relevant here [69]. The plant renewal process took six years, in contrast to the more typical two and a half years. This protracted license renewal process was due to the operator’s failure to include the community in the decision-making process [69]. This exclusion led to a law suit against the plant operators delaying licensing further. The failure to incorporate public participation is a clear violation of procedural justice norms. In so doing it also clearly fails to fairly distribute the risks and benefits by consulting those affected, thus raising concerns about distributive justice [69]. In addition, the operator’s failure to conduct risk assessment, risk management, risk decision-making, and risk distribution studies in accordance with best practices and principles of procedural and distributive justice it also necessarily violates the ethical obligations of nuclear energy [69]. It is obvious that as long as the authorities involved in regulating nuclear energy do not demonstrate their accountability and ethical responsibilities to public well-being, the problems of distributive justice and procedural justice will not be adequately addressed.

The overall evidence is that, in absence of clear indications of harm from low level radiation, the NRC ostensibly will renew licenses for existing nuclear power plants in operation. Given the absence of large scale epidemiological studies of populations within the 50-mile EPZ of U.S. reactors, there is little to stand in the way of near automatic license renewals of aging plants. Nevertheless, evidence from a series of European studies suggest that elevated childhood leukemia rates, among other diseases, are associated with proximity to reactor sites [28,29,30,31,32,33,34,35,36,37,38,39,40,41,42,43,44,45,46,48,70,71]. No such systematic studies have been conducted in the U.S.

## 3. Environmental Justice Issues in Nuclear Weapons Industrial Complex

While the commercial nuclear power program emerged out of the U.S. nuclear weapons program in the 1950s, the environmental health impacts of radioactive contamination in the U.S. date to the Manhattan Project, the accelerated federal program to build nuclear weapons in WWII. For the Manhattan Project and the subsequent large-scale program to build increasingly powerful and sophisticated nuclear weapons, the legacies of contamination are extensive, continuing, and difficult to remediate (e.g., [72]) In this section we extend our analysis of the environmental justice issues in nuclear technologies by examining landscape-scale radiation contamination as a result of nuclear weapons production and testing. We begin with a discussion of the health effects of several decades of uranium mining and milling on the Navajo nation. All contamination and nuclear waste issues discussed here involve transgenerational justice issues as some radioactive isotopes can remain lethal for tens of thousands of years and point to the substantial difficulties of being able to determine whether remediated federal weapons sites and waste deposition facilities will remain safe for millennia and not harm future generations [17,27]. The effects of uranium mining on the Navajo nation closely resemble the effects of uranium mining on local communities in other so-called third and fourth worlds [73]. While Navajo uranium supported the U.S. weapons program in the 1950s and 1960s, U.S. NPPs purchased 90% of their uranium from other countries including Canada, Australia, Russia, Kazakhstan, Namibia, and other countries in 2010 [74]. This demonstrates that maintaining nuclear power in the U.S. also concerns international justice issues.

### 3.1. Uranium Mining in Indian Country

The “uranium frenzy” began in the West in the 1940s as the U.S. ramped up its capacity to produce nuclear weapons providing a ready market for uranium ore [75]. While many sites on the Colorado Plateau were mined, the Navajo Nation—covering some 27,000 sq. mi. of Arizona, Utah, and New Mexico—became a center for mining and ore processing (milling). From 1944 to the 1980s uranium miners on the reservation produced more than 4 million tons of ore purchased by the federal government to use in the nuclear weapons program [76]. The exploitation of tribal resources and land for the U.S. weapons programs can be seen as a form of “nuclear colonialism”, wherein thousands of tribal members (and others) have been exposed to radiation in mines, nuclear fallout from weapons tests in Nevada, and have had their food and water resources contaminated by fallout and mining wastes, all with documented health effects [14,77]. By the 1980s when the demand for uranium declined, mines across the Navajo Nation were shut without remediation, leaving more than 500 known contaminated mine and mill tailing sites (and possibly hundreds more), poisoning communities, contaminating water, and strongly implicated in ongoing illness and disease among tribal members [77]. As a Navajo birth cohort study has recently shown, 27 percent of those tested had high levels of uranium in their urine, now more than 30–50 years after mines were closed [78]. That decades passed before the EPA began site remediation of mines and the clean-up of areas around homes speaks to the marginality of American Indians in federal environmental remediation efforts. Similarly, federal compensation for the health effects of uranium mining, nuclear testing, and community exposure only began in 1990, 45 years after the first nuclear explosion [10]. The federal government’s long delays in admission of harms done and reluctance to offer compensation is a common feature of nuclear injustices [13]. 

While uranium mining provided a few thousand Navajo with comparatively well-paying jobs, the health costs for those exposed in the mines, as well as thousands of others exposed by mine site, mill tailing, and groundwater contamination have been and continue to be extensive. Lung cancers began being documented among Navajo mine workers by the 1960s, the likely result of radon gas exposure in the mines although miners were never informed of the known risks. Lung cancer was virtually unheard of among Navajo and other Indian tribes prior to the advent of uranium mining but today the rates remain four times as high for miners as non-miners [77] in spite of no active reservation mining for decades (uranium mining on the Navajo Nation was banned by the tribal government in 2005 in response to the enduring environmental and health effects of previous mining). The incidence of kidney disease and other health complications among Navajo today are elevated and linked to drinking uranium-contaminated groundwater and living near unremediated mine sites [77]. The remediation costs for such extensive contamination runs in the billions of dollars, although a recent settlement between the Department of Justice with the Kerr-McGee Corporation netted $1 billion for the Navajo Nation for mine site clean-up and compensation to those sickened by mining and milling operations [79].

The experiences of the Navajo and other Indian tribes with uranium mining and processing as well as weapons testing exposure has clear and continuing environmental justice implications. These center on two key things: (1) the disregard of Indian communities’ health and well-being by the military and mining companies; and (2) the long delays between initial exposures and subsequent hazard mitigation and federal compensation programs for radiation victims. The federal Radiation Exposure Compensation Act (RECA) did not become law until 1990, 30–50 years after exposures from nuclear testing and mining, and after passage it has proved difficult for downwind and mining victims to receive benefits for their illnesses [75]. 

### 3.2. Federal Nuclear Weapons Centers

The contaminated federal research centers at Los Alamos, NM and Hanford, WA (other federal nuclear labs and production sites with extensive contamination issues include Savannah River (GA), Y-12 at Oak Ridge (TN), Rocky Flats (CO)—now closed but with significant contamination issues remaining—Fernald nuclear materials site (OH), and Lawrence Livermore Lab (CA)). All are active, difficult-to-remediate superfund sites and are further examples of the environmental and health risks that have been created in the process of producing nuclear weapons for the U.S. arsenal. Unlike tribal lands, these federal sites and numerous others are part of the national security state. As such they have lacked public oversight and have been cloaked in secrecy for much of their history [13]. 

Los Alamos National Laboratory (LANL) has a storied history as the center of the Manhattan Project and the production of the first nuclear weapons to be used in war. Established in 1943, it has a longer history of nuclear research and radioactive contamination than any other site in the world. Located in northern New Mexico on the Pajarito Plateau, it occupies the former homeland of the San Ildefonso Pueblo, gifted to the Manhattan Project, with the understanding that the lands would be returned at the end of WWII [12]. LANL sits in the center of more than a dozen Pueblo nations and within 50 miles of other Indian reservations including the Navajo and Jicarilla Apache [14]. Covering more than 43 square miles of forested uplands, it also encompasses many cultural sites sacred to Pueblo people, sites whose access is denied due to security at the weapons complex.

As typical of other federal nuclear research labs, LANL today has multiple sites of contamination from radiologic and chemical substances. Also typical of other nuclear labs, it has a history of intentional nuclear materials releases that have affected downwind communities [80]. While worker exposure to both low and high level radiation releases has been documented (see [13]), LANL’s use of conventional explosives combined with radioactive tracers (radiolanthanum or RaLa) to assess fallout patterns was kept secret from residents in surrounding communities until AEC and Department of Energy (DOE) documents on human radiation experiments were ordered declassified in 1994. There is substantial evidence of radioactive fallout from the RaLa tests in the 1950s and 1960s drifting over several Pueblo nations and other population centers east of LANL with no warnings ever being issued [80]. These fallout plumes were monitored by the Air Force to study dispersion patterns in anticipation of future nuclear tests elsewhere. As Masco [12] writes, “… the long-term effects of explosive testing and nuclear waste storage on the mountain raise fundamental questions about the safety of Pueblo lived spaces (p. 138)”. As with the Navajo and uranium mining, elevated rates of cancers in Pueblos adjacent to Los Alamos inevitably raise questions about cause given the near absence of cancers prior to the opening of the lab (e.g., [81]). That downwind communities here weren’t considered important enough to warn of potential risks, speaks to issues in recognition and procedural injustice typical of the nuclear weapons program. 

Among the legacies of 70 years of nuclear weapons research, testing, and production at LANL is an expanding nuclear dump site directly upstream/upwind of the San Ildefonso Pueblo and other communities. So-called Area G is a significant focus of concern both for Indian and Hispano communities in the area, and for anti-nuclear activists in New Mexico given the extensive store of highly radioactive materials and ongoing containment issues at LANL [12]. Clean-up here, as at other federal research sites, has been complicated by lack of federal funding and the lack of good records on what chemical contaminants and radioactive materials were dumped in pits prior to the 1980s [82]. Area G comprises LANL’s main dump site, now occupying 100 acres of mesa country adjacent to Pueblo lands: highly radioactive waste from decades of nuclear research and weapons fabrication, including plutonium-contaminated materials, is buried here. The site is nearing capacity, with 33 large burial pits, 220 deep shafts, and 4 trenches, the latter storing 7200 grams of weapons grade plutonium-239 in dry casks (p. 354 [12]). Above ground 16,000 barrels of radiation-contaminated liquid wastes await transportation to the Waste Isolation Pilot Project dump site in southern New Mexico (discussed below) [83]. 

Environmental health and justice concerns relate to the effects of possible exposure of downstream and downwind communities from leaks and airborne contamination at the site. Currently a tritium- and chromium-contaminated plume of groundwater is moving towards the San Ildefonso Pueblo from Area G, threatening to enter an aquifer that the Pueblo and other communities depend on for drinking water as well as for irrigation [84]. These risks are ongoing as are a litany of problems associated with clean-up of LANL’s multiple contamination sites. A DOE clean-up plan for LANL scheduled to be completed in December 2015 has substantially missed its deadline. Consistent with delays and cost overruns in other federal nuclear site clean-up programs (e.g., [85]) the lack of progress at LANL will see the program stretch out decades and costs escalate by billions of dollars [86]. Moreover, public endangerment remains in the absence of adequate site remediation by the DOE.

Another key site of the Manhattan Project, and one that poses perhaps even greater environmental safety and long-term health concerns for both surrounding communities and site workers, is the Hanford, WA site. The Hanford site covers 580 square miles of SE Washington, adjacent to a number of farming communities as well as 10 Indian reservations and is currently undergoing a very large scale site remediation process, estimated to ultimately cost $150 billion. Site clean-up has included removal of 20 tons of plutonium, debris from hundreds of irradiated structures, 2300 tons of spent nuclear fuel rods, thousands of tons of contaminated soil, and millions of gallons of highly contaminated waste water [85]. Hanford was a production center for weapons-grade plutonium for the Manhattan Project bombs and for the subsequent production of thousands of additional nuclear weapons. To that end, Hanford, until the 1980s, utilized nine nuclear reactors and other complex technologies to produce the plutonium cores for nuclear weapons. Doing so over its long operational life (1943–1988) has produced what is considered the single largest radiation and chemical contamination site in the Western Hemisphere [72]. The nine plutonium production reactors have been ‘cocooned,’ that is, encased in concrete, until the highly radioactive cores decay enough to be permanently disposed of sometime in the distant future [87]. While its role in the production of nuclear weapons has ceased, what remains at Hanford are multiple superfund sites of highly toxic wastes and a recent history of questionable success in containing and cleaning up the radiologic and chemical wastes [85]. 

Even while the plant was operating it had a dubious record of safety, with planned and unplanned releases of radioactive materials, accidents, worker exposures and radiation-related illnesses, as well as on-site dumping of radioactive liquids directly on the ground (450 billion gallons) creating numerous toxic groundwater plumes [72]. Evidence shows that residents in nearby communities have experienced persistent health issues ostensibly related to radioactive releases from the site over a period of decades, releases that were not announced nor warnings issued for [13]. Testimonies presented at public outreach meetings of the Advisory Committee on Human Radiation Experiments (ACHRE) in the 1990s contain a litany of stories on birth defects, autoimmune disorders, cancers, premature deaths, and thyroid disorders by those living near Hanford [80]. 

Hanford and AEC personnel monitored thousands of school children near the site in the 1960s to assess radionuclide body burdens on residents, as well as to assess levels of radioactive contamination of farmlands and crops. This was not out of concern for residents’ health but for better understanding the dispersal and bioaccumulation of radioactive materials in the ambient environments around Hanford (and other federal labs) (pp. 453–455 [13]). The incidence of cancers and other radiation related illnesses in nearby farm communities was so pronounced that the area became known as the “Death Mile” [72]. In 1986, documents were released by Hanford showing a multi-decadal history of leaks, intentional releases, and accidents. Over its years of operation more than 25 million curies of radionuclides were secretly released into the air and water around Hanford, more than that released at the Three Mile Island partial meltdown disaster (p. 147 [88]).

The most pressing problem at Hanford is 56 million gallons of highly unstable chemically- and radioactively-contaminated waste water stored in large underground tanks. At least one million gallons of waste water has leaked out of the tanks and has entered the Columbia river [72]. Given that a number of tribes in the area are permitted by treaty to harvest fish in the river, radioactive and chemical contamination ingested through fish consumption is a significant health concern for local Indian nations [88]. The larger issue is that the clean-up of Hanford poses substantial risks of radiological and chemical contamination and the site cleanup has been plagued by delays, cost overruns, cover-ups, and law suits by former workers. Further, a recent report verifies that the new waste treatment plant design is deeply flawed and subject to potential explosions and the release of nuclear and chemical materials [89]. 

### 3.3. Disposing of High Level Radioactive Waste

According to U.S. NRC, high-level wastes include (1) spent (used) reactor fuel and (2) waste materials remaining after spent fuel is reprocessed [90]. The latter type of high-level wastes is generated by both military nuclear reprocessing programs and commercial reprocessing operations. It is necessary to include both these waste streams in any high-level radioactive waste disposal plans [90]. Given the growing volume of high-level wastes nationally, commercial nuclear reactors and nuclear weapons programs have a common need: sites for permanent and safe storage of highly radioactive military waste and spent fuel rods. For high-level nuclear waste, the Nuclear Waste Policy Act Amendments (NWPAA) of 1987 specified one site for deep geologic burial, Yucca Mountain at the Nevada Test Site [91]. The NWPAA eliminated all other sites from consideration, shifting the environmental burdens of thousands of tons of spent fuel rods and related waste on the state of Nevada and Indian nations in proximity to Yucca Mountain [14,17,91]. No eastern site was ever under consideration although the majority of nuclear power plants are east of the Mississippi, raising significant procedural and distributive justice issues [92]. That Nevada has no nuclear power plants and has already borne substantial environmental burdens from decades of nuclear testing and fallout, points to pronounced inequities in terms of environmental health burdens vs. benefits [91]. 

Since taking office in 2009, the Obama administration announced that Yucca Mountain was no longer the presumed solution to the nation’s radioactive waste problem and stopped funding the program [91], although it remains a federal mandate. As with other federal nuclear projects, site assessment studies for a deep burial site have been plagued by falsified documents, massive cost overruns in the multiple billions of dollars, and delays already on the order of decades [14]. With the defunding of Yucca Mountain, the U.S. is left with no specific site for permanent safe storage of high-level waste.

In an effort to keep Yucca Mountain alive in May 2016 U.S. NRC completed and issued an Environmental Impact Statement supplement. While completion of adjudicatory hearing is necessary before making a decision on licensing, the site still remains suspended [93]. In January 2010, a Blue Ribbon Commission (BRC) was established by President Obama with a primary goal to develop a safe, long-term solution to managing the nation’s nuclear waste and spent nuclear fuel other than Yucca Mountain [94]. Two years later, the BRC recommended consideration of eight key elements in their first report including the prompt development of one or more geologic disposal facilities and other consolidated storage facilities [94]. U.S. DOE is responsible for constructing permanent sites whereas U.S. NRC is the regulator for design and operation of the facilities [93]. In January 2013, the U.S. DOE developed a framework to implement the BRC’s recommendations in the next 10 years in their report, Strategy for the Management and Disposal of Used Nuclear Fuel and High-Level Radioactive Waste [95]. The plan, which requires the consent of potentially impacted communities, includes constructing a pilot interim storage facility by 2021, and to advance toward the siting and licensing of a larger interim facility by 2025. It also includes plans for the site characterization of new repository sites to facilitate the availability of a geologic repository by 2048 [95]. The upshot is that even in this new plan the future of the permanent geologic repositories is still clouded with uncertainties at least for next three decades, while spent fuel rods continue to accumulate at commercial reactors.

These new plans notwithstanding, in the absence of a permanent site, highly radioactive spent fuel rods accumulate at commercial reactor sites, now estimated at around 70,000 tons [17]. On-site storage of plutonium-dense fuel rods in poorly secured commercial sites are a significant safety issue, given security concerns over terrorists acquiring nuclear materials to produce a “dirty bomb”—a device to spread nuclear contamination in a population center using conventional explosives. Indeed, recently the NRC has actually changed reactor security policies in such a way as to ostensibly reduce plant security against potential terrorist attacks (see [96]). 

The second nuclear waste geologic burial site, the Waste Isolation Pilot Project (WIPP) was opened in the 1990s and accepted military/research lab nuclear waste (transuranic waste), much of it coming from LANL’s Area G. (The WIPP site uses deep burial in hollowed out salt domes, and wastes are buried in stainless steel canisters. Over time the salt domes are expected to collapse entombing the waste.) The WIPP site was one of several sites evaluated in the 1970s that used hollowed out salt domes to store nuclear waste (other than fuel rods) underground. Concerns over water ingress and other issues delayed the opening of WIPP for more than two decades [92]. The WIPP site opened in the 1990s and began accepting waste from LANL, Hanford, and other federal sites in spite of strong state opposition [12]. However, after a period of relatively routine operation, in 2014 an improperly packed canister of radioactive waste from LANL caught fire underground, exposing workers to radiation and resulting in the venting of radioactive materials into the atmosphere (for information on the leak see [97]). It also revealed serious problems with how LANL was documenting and tracking radioactive waste to the site. With Yucca Mountain defunded and the WIPP closed pending extensive review of site safety and waste handling procedures, nuclear waste accumulates at reactor sites and clean-up at federal weapons sites is further delayed.

The primary concern of activists, tribes, and communities opposing these two sites is the sheer volume of nuclear waste that will traverse highways and railways through population centers in transit from nuclear reactors and nuclear weapons sites [12]. New DOE plans referenced above would still require the large scale movement of wastes through population centers. Given the recent history of oil train accidents in North America, legitimate concerns exist as to the environmental and human health consequences should a highway or rail accident result in the release of highly toxic radioactive material in a population center. While the DOE asserts that it is “impossible” for canisters containing highly radioactive materials to rupture, the recent fire and container breach at the WIPP site suggests otherwise. Currently, with no site open to accept high level or transuranic waste, nuclear waste transportation safety issues are temporarily reduced. Of course, Yucca mountain remains a federal mandate under the NWPAA, however ill-advised the location appears in site characterization studies [17]. The tail end of the nuclear fuel cycle—specifically permanent, safe burial—remains an unsolved technical problem, a deeply controversial political issue, and a significant transgenerational environmental justice concern.

## 4. Conclusions

This study has argued that nuclear power plants, uranium mining, and waste disposal raise a suite of justice issues including distributive, procedural, recognition and intergenerational justice issues. Moreover, these issues are transnational in scope and scale. In U.S., there are substantial uncertainties regarding the health effects of NPPs on the more than 87 million people residing within a 50-mile radius of a commercial reactor. These concerns are further complicated by the history of secrecy and the suppression of public participation in any nuclear decision-making by the NRC and DOE. Further, what participation is available is circumscribed by strict and self-serving procedural rules. Given the culture of the DOE and the NRC, and before it the AEC, there would appear a strong tendency of the promoters of nuclear energy to deny any potential health and environmental risks. Indeed, as discussed above, the lack of public discussion of emergency preparedness at NPPs illustrates this culture of minimizing risk and not raising public concerns or worries over the potential for accidents. The NRC has withheld nuclear power plant emergency plan documents systematically due to security concerns and has ignored comments by the public to improve plans. This same logic characterized the exposure of civilian populations to nuclear testing fallout: people were not informed of risks so as to not worry them [14]. Once radiation related diseases began in tribes and downwind communities after a two-decade latency, then a process of denial of responsibility by federal agencies ensued [9]. In a recent development, hundreds of U.S. sailors taking part in rescue efforts after the Fukushima’s accident have developed rare cancers, blindness, birth defects, and two deaths, leading to law suits against the Japanese nuclear power company [98]. 

Further procedural and recognition justice issues are associated with the nuclear weapons program and the sheer volumes of hazardous and poorly documented waste six decades of weapons production has produced at sites like LANL and Hanford. As weapons production is part of the national security state, it is highly secretive and public disclosures of risk typically come decades after exposures of civilian populations, if they come at all [13]. While federal programs like RECA belatedly became available for miners, downwind communities, and workers at federal weapons sites, the compensation has been typically limited, particularly in light of the long-term health care costs associated with diseases produced by radiation exposure [10]. Uranium mining has clearly related justice issues since it is not only workers who are exposed but their homes and communities also become contaminated [73]. That miners were never informed of the risks is further evidence of the disregard for the well-being of workers, often members of marginalized ethnic groups, by the nuclear industry. Of course, such experiences are not limited to North America: the experiences of uranium miners in African countries and other third world nations also illustrate the high environmental health costs and other international justice issues related to nuclear industries [73,74]. 

The continuing proliferation and operation of nuclear power plants globally and the waste they generate, constitutes substantial justice concerns for those living in proximity to nuclear sites and transportation corridors used for moving fuel and nuclear waste. China, for example, is on a crash course to build 40 new reactors in the next five years, a significant concern given their history of devastating industrial accidents [99]. Given the serious uncertainties over future high-level waste sites and the current poorly secured storage of highly radioactive spent fuel rods at reactor sites, the justice and environmental safety concerns raised here are significant. These currently unresolved problems are handed down to the coming generations to find solutions to (and pay for) producing substantial intergenerational injustice issues. Further, these issues will persist whether nuclear generation continues or countries go nuclear free: the waste is dangerous to all life for millennia.

Beyond the health risks of nuclear weapons production and NPPs, are the justice implications of the sheer costs of nuclear technologies particularly when the costs of waste disposal, contamination remediation at federal sites, and the decommissioning and entombment of NNPs at the end of their service life are calculated. That the radioactive components of a reactor as well as the spent fuel rods have to be securely stored for tens of thousands of years with limited risk to future generations, is on orders of magnitude different than any other industrial technologies. Given those cradle-to-grave environmental, health, and safety costs of nuclear power, renewable wind and solar technologies would appear to have major advantages both from a cost per kilowatt and for the lack of long-term health and safety risks to those in proximity and to future generations. That radioactive wastes and nuclear weapons and reactor technologies are potentially mutagenic, teratogenic, and carcinogenic, the potential multigenerational health risks are substantial. Subsequent generations will have to deal with highly radioactive wastes with technologies that currently don’t exist, revealing how nuclear technologies shift health, environmental, and financial burdens into the future, hiding their real costs and masking the procedural and recognition justice implications.

While there is periodic talk of a “nuclear renaissance” with smaller, cleaner, and safer reactors, the diseconomics of nuclear energy with the proliferation of low cost natural gas has utilities in the U.S. and Europe closing down expensive to operate reactors and switching to gas fired plants and a portfolio of renewable sources [100]. Indeed, in a recent white paper, Cooper argues that the diseconomics of nuclear power in comparison to both gas and renewables (solar and wind) is such that nuclear operators are poised to retire plants early given the expenses of keeping aging plants operating. While nuclear reactors’ putative zero carbon emissions are argued by some to be a necessary part of an effort to reduce carbon dioxide emissions, renewables offer more cost effective solutions without the intractable hazardous waste issues [100]. Of course the claim of zero carbon emissions ignores the emissions of the entire fuel cycle. In a post-Fukushima world, the expansion of nuclear power generation in the U.S. would require major government subsidies to overcome its economic and environmental hazards disadvantages. But given the current conservative drive both to cut federal spending and to deny the significance of anthropogenic climate change such a state centered approach to supporting nuclear expansion in the name of greenhouse gas reduction seems farfetched at best. It can also be argued that given the economic inefficiencies of nuclear power and the very high startup costs, far greater carbon savings can be found elsewhere. The global meat industry produces more greenhouse gases than the entire U.S., pointing to how a simple change in diet could reduce greenhouse gas emissions far more effectively than building billions of dollars’ worth of new reactors with their attendant risks [101].

What steps could be taken to begin to resolve some of the above discussed justice issues? To overcome all types of environmental justice issues, it is imperative for all key stakeholders including nuclear regulatory agency to take accountability and responsibility in carrying out activities in risk evaluation, risk decision-making, and risk management regarding nuclear power and radiation [69]. This requires full disclosure and public right-to-know principles and full democratic procedures in all nuclear issues, even those involving the military [27]. As long as the public is excluded by “national security” concerns and by government agencies relying on nuclear expert knowledge and self-serving rules that favor commercial interests over public well-being, justice will be elusive. Given the history of secrecy and denial in the U.S. over nuclear technology risks and impacts [14] whether a more just approach could be developed remains unclear. Clearly, phasing out of nuclear energy and nuclear weapons technologies, with their centralized and authoritarian tendencies [102] (as many European countries have initiated) is a positive step that responds to public opinion. Likewise, planning for high-level waste storage must involve democratic procedures and full consultation with those people and places that will be most affected. To do otherwise will repeat a history of nuclear injustice.

## Figures and Tables

**Table 1:** Demographic composition according to area, sorted by distance from any one of the 61 commercial NPPs, based on 2010 American Community Survey data.

2010	0–10	%	11–20	%	21–30	%	31–40	%	41–50	%	0–50	%	Outside	%
Tract (in thousand)	0.9	4	3	14	6	27	7	34	4	21	22	100	51	100
Tract area (sq. mile in million)	0.02	6	0.05	19	0.08	27	0.09	31	0.05	17	0.29	100	3.51	100
Total population (in million)	3.8	4	12.9	15	24.0	27	29.4	34	17.3	20	87.5	100	216.5	100
White (in million)	3.1	83.2	10.3	79.9	17.7	73.6	20.1	68.2	11.0	63.3	62.2	71.1	162.7	75.2
Black (in million)	0.4	10.6	1.6	12.2	4.0	16.8	5.5	18.7	3.6	20.9	15.1	17.3	22.8	10.6
Asian (in million)	0.1	2.0	0.3	2.7	0.8	3.3	1.5	5.0	1.2	6.8	3.8	4.4	10.8	5.0
Native American (in million)	0.0	0.3	0.0	0.3	0.1	0.3	0.1	0.4	0.1	0.3	0.3	0.3	2.2	1.0
Others (in million)	0.1	3.9	0.6	4.9	1.5	6.1	2.3	7.8	1.5	8.7	6.0	6.9	17.9	8.3
Hispanic (in million)	0.3	8.2	1.3	10.0	3.0	12.4	4.3	14.7	2.6	15.2	11.5	13.2	36.2	16.7
Color (in million)	0.8	22.3	3.4	26.4	8.1	33.9	11.7	39.7	7.7	44.4	31.8	36.3	75.6	34.9
